# Efficacy and Safety of GLP-1 Receptor Agonists in Patients With Metabolic Dysfunction-Associated Steatotic Liver Disease: A Systematic Review and Meta-Analysis of Randomized Controlled Trials

**DOI:** 10.7759/cureus.71366

**Published:** 2024-10-13

**Authors:** Basile Njei, Yazan Al-Ajlouni, Samira Y Lemos, Derek Ugwendum, Prince Ameyaw, Lea-Pearl Njei, Sarpong Boateng

**Affiliations:** 1 Department of Medicine, Yale School of Medicine, New Haven, USA; 2 Department of Medicine, New York Medical College, New York, USA; 3 Department of Diabetes and Endocrinology, Yaoundé General Hospital, Yaoundé, CMR; 4 Department of Internal Medicine, Richmond University Medical Center Affiliated with Mount Sinai Health System and Icahn School of Medicine at Mount Sinai, Staten Island, USA; 5 Department of Internal Medicine, Bridgeport Hospital, Yale New Haven Health, Bridgeport, USA; 6 Department of Biological Science, University of Maryland Baltimore County, Baltimore, USA; 7 Department of Medicine, Bridgeport Hospital, Bridgeport, USA

**Keywords:** cardiovascular risk factors, glp-1 receptor agonists, hepatic outcomes, metabolic dysfunction-associated steatotic liver disease, weight management

## Abstract

Metabolic dysfunction-associated steatotic liver disease (MASLD) poses a major global health challenge. glucagon-like peptide-1 receptor agonists (GLP-1RAs) have shown potential therapeutic benefits for MASLD patients, including improvements in liver function, inflammation, and fibrosis. This study aims to systematically review and meta-analyze randomized controlled trials (RCTs) to evaluate the efficacy and safety of GLP-1RAs in MASLD patients, focusing on hepatic outcomes, cardiovascular outcomes, anthropometric measurements, and mortality. Following PRISMA guidelines, a comprehensive database search was conducted to include RCTs assessing GLP-1RAs' effects on MASLD. Quality assessment was conducted using the Revised Cochrane Risk of Bias tool. Our meta-analysis used a random-effects model, calculating standardized mean differences for continuous outcomes to determine the agents' efficacy and safety. Additionally, funnel plots were generated to assess publication bias, ensuring the integrity of our meta-analytical findings. The review included 27 trials, revealing GLP-1RAs significantly improved hepatic function markers (alanine aminotransferase, aspartate aminotransferase, gamma-glutamyl transferase, and liver fat content) and cardiovascular risk factors (fasting blood sugar, HbA1c levels, lipid profiles). Additionally, GLP-1RAs were associated with significant reductions in body weight, BMI, subcutaneous fat, and waist circumference. GLP-1RAs demonstrate a promising therapeutic role in managing MASLD, offering benefits that extend to improving liver function, mitigating cardiovascular risk, and promoting weight loss. Further research is needed to confirm these findings and optimize GLP-1RAs' usage in MASLD treatment.

## Introduction and background

Metabolic dysfunction-associated steatotic liver disease (MASLD), a type of steatosis that is caused by factors other than excessive alcohol use, poses a rising global health challenge, affecting approximately 25% of adults worldwide [1]. The increasing prevalence highlights the imperative to grasp MASLD's profound implications within the broader context of global health. Beyond its sheer ubiquity, MASLD intricately links with cardiovascular events, insulin resistance, and type 2 diabetes mellitus [2,3]. Recent research reveals its association with an elevated risk of cardiovascular events [4-9]. Simultaneously, MASLD's integral role in the complex interplay of insulin resistance and the development of type 2 diabetes mellitus is gaining recognition [10,11]. Literature suggests a bidirectional causal relationship between MASLD and diabetes mellitus, emphasizing the potential influence of insulin resistance in MASLD progression [12,13]. As MASLD's impact extends across these interconnected domains, addressing this multifaceted condition becomes pivotal for comprehensive global health management. Moreover, with the evolving understanding of the MASLD-insulin resistance relationship, exploring therapeutic interventions for insulin resistance or diabetes emerges as a compelling avenue, although the exact implications remain to be fully elucidated.

In the context of diabetes management, glucagon-like peptide-1 receptor agonists (GLP-1RAs) have emerged as a pivotal therapeutic option, presenting multifaceted benefits beyond glycemic control. Ongoing research consistently highlights their efficacy in improving blood glucose control, preserving beta-cell function, renoprotective effects, inducing weight loss, and enhancing insulin sensitivity [14-17]. Moreover, GLP-1RAs extend their impact beyond glycemic control by addressing various metabolic disorders, including the improvement of lipid profiles and reduction in blood pressure [14,17,18]. Within the context of MASLD, GLP-1RAs proved promising in ameliorating hepatic steatosis, inflammation, and fibrosis, emphasizing their anti-inflammatory and anti-fibrotic properties [19-21]. These hepatic effects involve indirect pathways, such as adaptations in plasma insulin and glucagon concentrations, improvements in hepatocyte mitochondrial function, and enhanced hepatic insulin sensitivity. Despite conflicting results in the literature, recent evidence may suggest that hepatocytes lack direct GLP-1 receptors [22-24]. Collectively, the diverse benefits of GLP-1RAs position them as valuable therapeutic agents for comprehensive diabetes management, offering potential avenues for addressing hepatic manifestations in MASLD.

While existing literature acknowledges the efficacy of GLP-1RAs in managing MASLD, there remains a gap in our understanding that necessitates further exploration. Presently, systematic reviews on this topic exist; however, many encompass a broader spectrum by incorporating various medications and patients with diverse diseases [25-28]. This inclusivity, while informative, lacks the specificity needed for a focused evaluation of GLP-1RAs' efficacy in MASLD treatment. Therefore, there is a clear need for a comprehensive review specifically tailored to assess multiple outcomes among MASLD patients. Moreover, given the evolving landscape of evidence in the literature, there is a compelling need for updated reviews that can synthesize and incorporate the latest findings.

Upon those bases, this paper aims to conduct a comprehensive systematic review and meta-analysis focused on the efficacy and safety of GLP-1RAs in the context of MASLD. By synthesizing existing evidence, our objective is to provide a detailed assessment of the multifaceted benefits of GLP-1RAs, extending beyond glycemic control to encompass their potential impact on hepatic manifestations in MASLD. Recognizing the current gaps in the literature, particularly the lack of focused reviews on GLP-1RAs within the MASLD population, this study endeavors to offer a tailored examination of outcomes specific to MASLD patients. Furthermore, our investigation seeks to address the limitations of previous systematic reviews, often incorporating diverse medications and patient cohorts, by homing in on the unique therapeutic potential of GLP-1RAs. Anticipating the dynamic nature of emerging evidence, our review aims to provide an updated and nuanced perspective on the subject. The potential implications of our findings extend beyond the realms of diabetes management, offering a novel perspective on the role of GLP-1RAs in the broader landscape of MASLD treatment.

Of note, our article was previously posted to the Authorea preprints server on May 16, 2024. 

## Review

Methods

Search Strategy

This meta-analysis adhered to the rigorous standards outlined in the Preferred Reporting Items for Systematic Reviews and Meta-Analyses (PRISMA) guidelines [29]. To identify relevant studies, a comprehensive search was conducted across multiple databases, including PubMed, CINAHL, Medline, Embase, Web of Science, and PsychINFO. The search encompassed articles reporting the efficacy of GLP-1RAs in patients with Metabolic Associated Fatty Liver Disease (MASLD). The search period spanned from the inception of the databases through January 10, 2024.

A systematically developed keyword strategy, collaboratively refined by the coauthors, was employed for the database search. The details of the keyword strategy are provided in Appendix A. This strategy aimed to capture studies investigating the impact of GLP-1RAs on MASLD patients, including mortality, cardiovascular outcomes, and hepatic outcomes.

Eligibility and Selection Criteria

The study selection process adhered to predetermined eligibility criteria to ensure the relevance and reliability of included studies. Inclusion criteria encompassed studies conducted in English, reporting primary outcomes related to the efficacy, safety, or treatment outcomes of GLP-1RAs in patients with MASLD. Only randomized controlled trials (RCTs) were considered, with a focus on adult participants (18+ years old) diagnosed with MASLD. Full-text availability and peer-reviewed status prior to final publication were additional criteria for inclusion.

Exclusion criteria included studies lacking peer review, those with inaccessible full texts or written in languages other than English, and non-RCT study designs such as literature reviews, case reports, case series, or those conducted in animal models. Pediatric populations and studies unrelated to MASLD or GLP-1RAs were also excluded. Appendix B provides detailed information on the criteria, ensuring transparency and facilitating a comprehensive understanding of the selection process.

Data Extraction

Relevant data extraction was conducted by two independent authors using a predefined template. This template systematically captured essential information pertaining to study characteristics, encompassing sample size, country of origin, and study design. Qualitative extraction was employed for baseline information, ensuring a comprehensive inclusion of relevant details.

For therapeutic regimen and treatment outcomes, a quantitative approach was adopted. Specifically, the template included details on the therapeutic regimen of the treatment methods and comprehensive treatment outcomes. These outcomes comprised changes in body composition, metabolic parameters, cardiovascular outcomes (e.g., triglyceride, cholesterol levels), and hepatic outcomes (e.g., aspartate aminotransferase [AST] and alanine aminotransferase [ALT] levels).

In instances where mean values and standard deviations for continuous variables were not provided, estimates were derived using established formulas [30,31]. To enhance the reliability of the data, a rigorous validation process was implemented. An independent third author conducted blind checks of the data, and any disparities were resolved through consensus among the authors. This dual approach, combining qualitative extraction for baseline information and quantitative extraction for specific outcomes, ensured a thorough and accurate compilation of data for subsequent meta-analysis.

Quality Assessment

The quality assessment of included RCTs was performed using the Revised Cochrane Risk of Bias tool (RoB2) developed by the Cochrane Collaboration and modified to ensure standardized scoring [32]. This tool is recognized for its comprehensive evaluation of key domains, ensuring a robust assessment of the methodological quality of each study.

Two independent reviewers conducted the quality assessment, with any discrepancies resolved through discussion and consensus. The RoB2 tool was applied to appraise the risk of bias in the following domains: randomization process, deviations from intended interventions, missing outcome data, measurement of the outcome, and selection of the reported result. Each domain was evaluated for low, some concerns, or high risk of bias, providing a nuanced understanding of the overall study quality.

Statistical Analysis

For the quantitative synthesis, values of standardized mean differences were computed for continuous outcomes, providing a standardized metric for the comparison of effect sizes across diverse studies. The statistical analyses were conducted using R version 4.3.1 (R Foundation for Statistical Computing, Vienna, Austria), leveraging specialized packages such as MetaInsight and Meta-Mar for modeling and visualization of the synthesized data [33]. The comprehensive approach included the generation of forest plots, allowing for a visual representation of individual study effect sizes and their overall combined effect. Additionally, funnel plots were constructed to assess publication bias, offering insights into the symmetry of the distribution of study effects. The meta-analysis employed a random-effects model to account for potential variability across studies. Heterogeneity assessment was conducted using both the chi-square and I-square tests. The chi-square test determined the presence of heterogeneity, while the I-square test quantified the degree of heterogeneity.

Ethical Approval and Funding

For this systematic review, ethical approval is considered unnecessary since the data are derived from pre-existing literature. The studies included in this review underwent ethical review and clearance by their respective primary investigators before data collection. Additionally, this work received no funding.

Results

Study Selection

**Figure 1:**
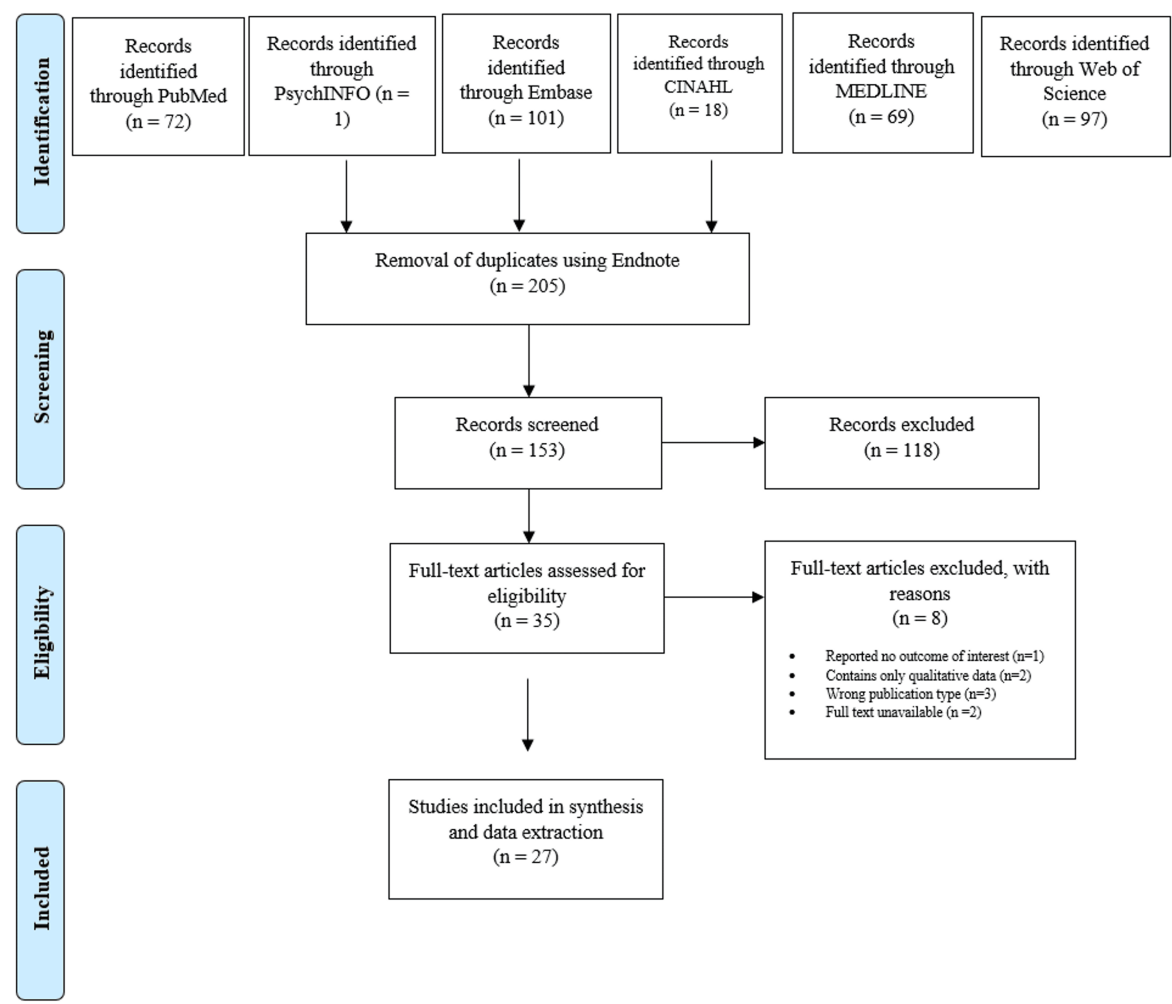
PRISMA study selection flow diagram. PRISMA, Preferred Reporting Items for Systematic Reviews and Meta-Analyses

In the systematic review, our search strategy across six databases (Web of Science, MEDLINE, CINAHL, Embase, PsychINFO, and PubMed) resulted in the identification of 358 records. After the removal of 205 duplicates using Endnote, 153 records were screened, leading to the exclusion of 118 records. Of the 35 full-text articles assessed for eligibility, eight articles were excluded for various reasons including lack of relevant outcomes, qualitative data only, incorrect publication type, and unavailability of full text, leaving 27 studies included in the synthesis and data extraction. A total of 27 trials were included, with four trials reporting two analyses (e.g., different control groups) [27,34-36]. Figure 1 shows the study selection process according to the PRISMA guidelines.

Characteristics of Included Studies

The included RCTs encompassed a diverse population of patients with MASLD. The characteristics of the included studies are summarized in Table 1. The included studies reveal significant diversity in design, geography, and patient demographics, investigating the impact of GLP-1 agonists on MASLD across various global locations including Denmark, the UK, Japan, the USA, China, Singapore, India, Greece, Canada, and more. These studies encompass a range of RCTs, open-label, and retrospective analyses, with patient populations varying from small pilot studies to larger multicenter trials. The interventions primarily focused on GLP-1 agonists such as semaglutide, liraglutide, exenatide, and dulaglutide, with durations ranging from 12 to 72 weeks.

**Table 1:** Baseline characteristics of all included studies investigating the effect of GLP-1 among MASLD patients. ALT, alanine aminotransferase; ASAT, aspartate aminotransferase; AST, aspartate aminotransferase; BMI, body mass index; CAP, controlled attenuation parameter; DPP-4i, dipeptidyl peptidase-4 inhibitors; FIB-4, fibrosis-4 index; GLP-1, glucagon-like peptide-1; HbA1c, hemoglobin A1c; hsCRP, high-sensitivity C-reactive protein; IHCL, intrahepatic lipid content; IHF, intrahepatic fat; IHL, intrahepatic lipid; LDL-C, low-density lipoprotein cholesterol; LFC, liver fat content; MASLD, metabolic associated steatosis liver disease; MRE, magnetic resonance elastography; MRI, magnetic resonance imaging; MRI-PDFF, MRI-proton density fat fraction; MRS, magnetic resonance spectroscopy; NAFLD, non-alcoholic fatty liver disease; NASH, non-alcoholic steatosis hepatitis; SAT, subcutaneous adipose tissue; pGDM, previous gestational diabetes mellitus; T2DM, type 2 diabetes mellitus; VAT, visceral adipose tissue; VCTE, vibration-controlled transient elastography

Study/year	Location	Study type	Total patient population/ sample size	Demographics	Drugs used/patient group/treatment and control	Intervention/ duration dose,	Additional diagnosis for patients	Diagnostic method	Adverse events	Outcomes/main findings	Limitations if any
Flint et al., (2021) [37]	Denmark	Randomized, double-blind, placebo-controlled trial	Total 67 with semaglutide 0.4 mg (n = 34) and placebo (n = 33)	Subjects aged 18-75 years, with a BMI of 25-40 kg/m^2^, liver stiffness of 2.50-4.63 kPa measured by MRE and >4.0 kPa measured via vibration controlled transient elastography (VCTE) (FibroScan®, EchoSens, Paris, France)	Subcutaneous semaglutide 0.4 mg once daily or placebo for 72 weeks	72 weeks	No	MRE	Decreased appetite, Diarrhea, nausea, vomiting, nasopharyngitis, constipation, abdominal pain upper, dizziness, flatulence, eructation, headache, fatigue, early satiety	Reductions in liver steatosis were significantly greater with semaglutide, and more subjects achieved a ≥30% reduction in liver fat content with semaglutide at weeks 24, 48, and 72. Decreases in liver enzymes, body weight, and HbA1c were also observed with semaglutide.	Few subjects (15% overall) had advanced disease/a high degree of liver stiffness (≥3.64 kPa) at baseline, which may have made it difficult to detect a decrease in liver stiffness.
Newsome et al., (2021) [38]	UK	Placebo-controlled trial	320 patients	18 to 75 years of age (20 to 75 years of age in Japan), with or without T2DM, and a BMI of >25 kg/m^2^ at screening.	Once-daily subcutaneous semaglutide at a dose of 0.1, 0.2, or 0.4 mg or corresponding placebo	72-week	T2DM	Biopsy-confirmed NASH and liver fibrosis of stage F1, F2, or F3	Gastrointestinal disorders (nausea, constipation, decreased appetite, vomiting, and abdominal pain)	An improvement in fibrosis stage occurred in 43% of the patients in the 0.4-mg group and in 33% of the patients in the placebo group	Interobserver variability, as well as a lack of long-term clinical outcomes
Eguchi et al., (2015) [39]	Japan	Pilot study	27	Age (years) 54.1 ± 12.3, BMI 31.6 ± 4.9 kg/m^2^, sex (female/male) 16/11	Liraglutide at 0.9 mg/body per day for 24 weeks	24 weeks	No	Liver biopsy	No	Treatment with liraglutide had a good safety profile and significantly improved liver function and histological features in NASH patients with glucose intolerance	Open-label design, short treatment period and the small numbers of patients
Loomba et al., (2023) [40]	USA	Double-blind, placebo-controlled phase 2 trial	71	Age 59.5 years (SD 8.0) and mean BMI of 34.9 kg/m², 49 (69%) patients were female and 22 (31%) were male.	Once-weekly subcutaneous semaglutide 2.4 mg or visually matching placebo	48 weeks	T2DM	Biopsy	Gastrointestinal adverse events were mild-to-moderate transient nausea, diarrhea, and vomiting	Semaglutide did not significantly improve fibrosis or achievement of NASH resolution versus placebo.	Relatively small size
Armstrong et al., (2015) [41]	UK	Multicenter, double-blinded, randomized, placebo-controlled phase 2 trial	26+26	Age 50 -52 years,	Subcutaneous injections of liraglutide (1∙8 mg daily)	48 weeks	T2DM	Liver biopsy	Gastrointestinal disorders	Liraglutide was safe and well tolerated, and led to histological resolution of non-alcoholic steatohepatitis, warranting extensive, longer-term studies	Sample size
Feng et al., (2017) [42]	China	Randomized trial	87	Age 18–70 years, HbA1c 7.0–14.0%, BMI 20–38 kg/m^2^	For the metformin group (n = 31), the treatment regimen started with 250 mg three times daily in the first week, increased to 500 mg three times daily in the second week, and then adjusted to 1,000 mg twice daily from the third week until the study ended. In the gliclazide group (n = 31), participants began with a 30 mg dose before breakfast, which was gradually increased to a maximum of 120 mg per day to maintain fasting capillary plasma glucose levels below 7.0 mmol/L. The liraglutide group (n = 31) started with 0.6 mg per day in the first week, progressed to 1.2 mg per day in the second week, and reached 1.8 mg per day from the third week onwards until the study was completed.	24 weeks	Diabetes	Ultrasonography hepatic/renal [H/R] ratio	Appetite suppression, nausea, diarrhea, abdominal distension, and temporary rash at the injection site	Compared to liraglutide and metformin, gliclazide showed less improvement in liver function, smaller reductions in intrahepatic fat content and HbA1c levels, and resulted in less weight loss	The authors acknowledge that the sample size in this study was limited. Additionally, due to budgetary constraints, they did not employ 1H-MRS, which is recognized as the gold standard for evaluating intrahepatic fat.
Khoo et al., (2017) [43]	Singapore	Pilot randomized trial	24	Obese (BMI ≥ 30 kg/m^2^, mean weight 96.0 ± 16.3 kg) non-diabetic Asian adults	Liraglutide was started at 0.6 mg daily and increased by 0.6 mg weekly until 3 mg was reached at the end of 4 weeks and maintained for the next 22 weeks	26 weeks	No	ALT, AST, MRI	Gastrointestinal problems	Weight loss induced with liraglutide 3 mg daily is as effective as a structured lifestyle intervention for reducing severity of NAFLD in obese Asian adults	Hepatic steatosis and steatohepatitis, respectively
Khoo et al., (2019) [44]	Singapore	Randomized trial	30	Mean age 40.7 ± 9.1 years, BMI 33.2 ± 3.6 kg/m^2^, 90% male	liraglutide 3 mg daily and a group with energy restriction plus moderate-intensity exercise to induce ≥5% weight loss	26 weeks	No	MRI	Gastrointestinal adverse effects	Liraglutide was effective for decreasing weight, hepatic steatosis, and hepatocellular apoptosis in obese adults with NAFLD, but benefits were not sustained after discontinuation, in contrast with lifestyle modification	The authors noted the short duration of the study, small sample size, absence of a control group, and reliance on MRI and serum liver transaminases as surrogates for liver biopsy in assessing hepatic steatosis and steatohepatitis, respectively.
Liu et al., (2020) [45]	China	Randomized controlled multicenter clinical trial	76	Aged 18−70, BMI >24 kg/m^2^, glycated hemoglobin A1c (HbA1c) level between 7% and 10%	Subcutaneous exenatide (Byetta) 5 μg twice daily for 4 weeks and 10 μg twice daily for 20 weeks, or subcutaneous insulin glargine (Lantus) for 24 weeks	24 weeks	T2DM	Magnetic resonance spectroscopy, FIB-4 index	Hypoglycemia	Both exenatide and insulin glargine reduced LFC in patients with drug-naive T2DM and NAFLD; however, exenatide showed greater reductions in body weight, visceral fat area, liver enzymes, FIB-4, postprandial plasma glucose, and LDL-C	open-label trial design may have introduced bias, lack of individual assessment of dietary and exercise changes although dietary and exercise guidance have been given to patients.
o et al., (2014) [46]	China	Randomized study	60	age 20–70 years; BMI ≥ 28 kg/m^2^	The exenatide treatment group (n=30) were treated with exenatide and insulin glargine, and the intensive insulin therapy group (n=30) were treated with insulin aspart and insulin glargine for 12 weeks	12 weeks	T2DM	Abdominal ultrasound	Mild-to-moderate gastrointestinal side effects	Exenatide has a better hepatic–protective effect than intensive insulin therapy and perhaps represents a unique option for adjunctive therapy for patients with obesity, non-alcoholic fatty liver disease with elevated liver enzymes and T2DM	Only 60 subjects were recruited
Yan et al., (2019) [47]	China	Open-label, active-controlled, parallel-group, multicenter trial		Aged 30-75 years with T2DM and HbA1c levels between 6.5% and 10%	Subcutaneous liraglutide 1.8 mg once daily, oral sitagliptin 100 mg once daily, or subcutaneous insulin glargine at bedtime plus metformin for 26 weeks	26 weeks	T2DM	Clinically diagnosed NAFLD with MRI	Gastrointestinal disorders, headache, toothache	Combined with metformin, both liraglutide and sitagliptin, but not insulin glargine, reduced body weight, IHL, and VAT in addition to improving glycemic control in patients with T2DM and NAFLD	Lack of a placebo control was a weakness of our study, and the open-label trial design may have introduced bias
ng et al., (2020) [48]	China	Single-center, open-label, prospective, and randomized trial	60	Age of 18—70 years, glycosylated hemoglobin A1c (HbA1c) of 7.0-14%, Body mass index (BMI) of 20—35 kg/m^2^	For the liraglutide group (n = 30), the dosage was 0.6 mg/day during the first week, 1.2 mg/day from the second week until the conclusion of the study.	24 weeks	T2DM	1H-MRS on a 1.5 T whole-body MRI scanner	Gastrointestinal reactions include poor appetite, nausea, vomiting, acid reflux, abdominal pain, bloating, and diarrhea	Liraglutide promotes a larger decrease in the hepatic fat content and fetuin-A than pioglitazone in patients with T2DM and NAFLD	Sample size was small and specific to older adults with T2DM
Bi et al., (2014) [34]	China	Randomized study	33	52.7 ± 1.7 years of age, with a BMI of 24.5 ± 0.5 kg/m^2^, HbA1c 8.7 ± 0.2%	Exenatide, the initial dose was 5 mg twice daily and titrated to a maximum 10 mg twice daily at week 4 and maintained throughout the course of the trial	24 weeks	T2DM	MRS	Not reported	In patients with drug-naive T2DM, liver fat content can be significantly decreased irrespective of using exenatide, insulin, or pioglitazone therapy. Early metabolic control, including glycemic control and weight reduction, plays an important role in slowing progression of fatty liver in T2DM.	Smaller number of subjects that might weaken the statistical power
Guo et al., (2020) [35]	China	Randomized placebo-controlled trial	96	Age 30–60 years, BMI > 25 kg/m^2^ at screening	Subcutaneous injection of liraglutide at a starting dose of 0.6 mg 1/ day and increased by weekly forced titration to 1.8 mg	26 weeks	T2DM	Clinically diagnosed	Nausea, abdominal distension, diarrhea and rash	After 26 weeks of treatment, compared to the placebo group, in the liraglutide and insulin glargine groups, IHCL significantly decreased from baseline to week 26. SAT and VAT decreased significantly in the liraglutide group and in the insulin glargine group.	Liver histopathological biopsy was not used to evaluate the liver fat content of patients; Also, the number of patients included is still small (n = 91)
Kuchay et al., (2020) [49]	India	Open-label, parallel-group, randomized controlled trial	64	Age 48.1 ± 8.9 years, 45 males	Dulaglutide 0.75 mg by subcutaneous injection each week for 4 weeks, then 1.5 mg weekly for 20 weeks plus standard treatment for T2DM	24 weeks	T2DM	MRI-derived proton density fat fraction-assessed LFC of ≥6.0% at baseline	Gastrointestinal	Dulaglutide significantly reduces LFC and improves GGT levels in participants with NAFLD. There were non-significant reductions in PFC, liver stiffness, serum AST and serum ALT levels.	Authors did not use placebo in the control group
Smits et al., (2016) [36]	Netherlands	Randomized placebo-controlled trial	52	Age 62.7 ± 6.9 years, HbA1c 7.3 ± 0.7% or 56± 1 mmol/mol	Liraglutide 1.8 mg taken once daily in the evening.	12 weeks	T2DM	1H-MRS	Dizziness and daytime urinary frequency	Twelve-week liraglutide or sitagliptin treatment does not reduce hepatic steatosis or fibrosis in T2DM	Sample size was small, it is unlikely that a larger trial would have yielded different results given the minimal between-group differences
Tang et al., (2015) [50]	Canada	Randomized trial	35	Age 60.7 ± 16.1 years; BMI 31.3 ± 4.1 kg/m^2^	Liraglutide was started on 0.6 mg subcutaneously per day and increased by weekly forced titration to 1.8 mg or the maximal tolerated dose	12 weeks	T2DM	MRI	Nausea, diarrhea, vomiting, headaches	Improvements in the liver fat fraction and glycemia control were not significantly different from those in the liraglutide group.	The choice of MR rather than liver biopsy as the reference standard
Alkhouri et al., (2022) [51]	USA	A randomized, open-label phase II trial	108	Aged 18–75 years	Semaglutide plus cilofexor 30 mg (SEMA + CILO 30), semaglutide plus cilofexor 100 mg (SEMA + CILO 100), semaglutide plus firsocostat 20 mg (SEMA + FIR) or semaglutide plus cilofexor 30 mg plus firsocostat 20 mg (SEMA + CILO + FIR)	24 weeks	No	F2–F3 on biopsy, or MRI-proton density fat fraction (MRI-PDFF)	Grade 3 diarrhea and vomiting	Treatment resulted in additional improvements in liver steatosis and biochemistry vs. semaglutide alone	Trial enrolled small numbers of patients across 5 treatment groups, was open label, and lacked a placebo-controlled group
Gastaldelli et al., (2022) [52]	USA	Randomized, open-label, parallel-group, phase 3 SURPASS-3 trial	296	BMI of at least 25 kg/m², stable weight	Subcutaneous injection once per week of tirzepatide 5 mg, 10 mg, or 15 mg, or subcutaneous injection once per day of titrated insulin degludec	52 weeks	T2DM	MRI scan	Not reported	Tirzepatide showed a significant reduction in LFC and VAT and ASAT volumes compared with insulin degludec in this subpopulation of patients with T2DM in the SURPASS-3 study.	There were no biopsy results available, and therefore associations with histological changes cannot be established
Makri et al., (2022) [53]	Greece	Retrospective study	189	Age 18 years; patients with T2DM on metformin with or without other anti-diabetic medications; follow-up of 6-18 months	Within the DPP-4i group, the patients received sitagliptin (100 mg once daily), vildagliptin (50 mg twice daily), linagliptin (5 mg once daily), alogliptin (25 mg once daily) or saxagliptin (5 mg once daily). Within the GLP-1 RA group, the patients received exenatide (10 mcg twice daily), dulaglutide (1.5 mg once weekly) or liraglutide (1.2 mg once daily).	6-18 months	T2DM	Ultrasound	No	NAFLD ridge score was significantly decreased after the addition of GLP-1 RA in patients with T2DM	Data were prospectively recorded but retrospectively retrieved, which introduced limitations inherent to retrospective studies
Newsome et al., (2019) [54]	UK	Phase 2, randomized, double‐blind, multinational, placebo‐ and active‐controlled dose‐finding trial	957	Age range: 35‐65 years	semaglutide was given once daily for 52 weeks at subcutaneous doses of 0.05, 0.1, 0.2, 0.3 or 0.4 mg	52 weeks	T2DM, cardiovascular	NAFLD Fibrosis Score and Fibrosis 4 Index	Adverse cardiac event	Semaglutide significantly reduced ALT and hsCRP in clinical trials in subjects with obesity and/or T2DM	Neither trial enrolled subjects with confirmed NASH and histology data were not available
Ohki et al., (2012) [55]	Japan	Retrospective study	82	Age 54.2 (44.4–63.2), Male 61	Liraglutide was subcutaneously injected once daily 0.3mg for the first week, 0.6 mg for the next week, and finally up to the limit dose 0.9 mg if necessary. Sitagliptin was administered via oral route once daily 50 mg up to 100 mg if necessary. Pioglitazone was administered once daily 15 mg via oral route.	Liraglutide and sitagliptin was about 340 days and 250 days	T2DM	Clinically diagnosed NAFLD	No	Administration of liraglutide improved T2DM but also improvement of liver inflammation, alteration of liver fibrosis, and reduction of body weight	Difference in dosing period of each medicine, median dosing period of pioglitazone was about 1,200 days, the median dosing period of liraglutide and sitagliptin was about 340 days and 250 days
Fan et al., (2013) [56]	China	Research study	117	Mean age was 52.35 ± 11.83 years, 66 males and 51 females	Exenatide injection was administrated from week 1 to week 4 at 5 μg (bid), and from week 5 to week 12 at 10 μg (bid).	12 weeks	T2DM	Ultrasonography	No	Compared with metformin, exenatide is better to control blood glucose, reduces body weight and improves hepatic enzymes, attenuating NAFLD in patients with T2DM concomitant with NAFLD.	Not reported
Tian et al., (2018) [57]	China	Randomized study	127	Age in the range of 21–70 years	Liraglutide group received liraglutide at a dose of 0.6–1.2mg/day and the metformin group received metformin at a dose of 100–1,500 mg/day	12 weeks	T2DM	B-mode ultrasonic scanning	Not reported	Liraglutide was better than metformin in its ability to significantly decrease the ALT levels in patients with combined T2DM and NAFLD. Furthermore, liraglutide was more effective than metformin at ameliorating the severity of T2DM complicated with NAFLD, and produced its effects by alleviating liver inflammation and improving liver function	Not reported
Armstrong et al., (2016) [58]	UK	Randomized study	14	Adult age (18–70 years) and had a body mass index (BMI) >25 kg/m^2^	1.8 mg liraglutide or placebo	12 weeks	No	Liver biopsy	Not reported	Liraglutide reduces metabolic dysfunction, insulin resistance and lipotoxicity in the key metabolic organs in the pathogenesis of NASH.	Sample size limits statistical sub-group analysis
Savvidou et al., (2016) [59]	Greece	Open-label, randomized controlled intervention trial	127	The study sample included 49 males (38.6% of participants), with a mean age of 63.1 ± 7.5 years and a mean BMI of 32.9 ± 4.9 kg/m²	Exenatide was supplied through subcutaneous injections in the upper arm, thigh or abdomen with prefilled pens of 5 mg twice daily for the first 4 weeks and 10 mg (forced titration) twice daily thereafter. Patients were self-injected within 60 min before morning and evening meals.	24 weeks	T2DM	Liver biopsy	Redness at sites of injection (both groups), morning hypoglycemic events easily reversible by oral intake of juice (both groups), and mild abdominal discomfort after exenatide injections	Supplementation of exenatide to glargine insulin compared to standard insulin was: (i) effective in inducing weight loss, (ii) non-inferior in lowering HbA1c and, (iii) noninferior in increasing circulating adiponectin	NAFLD patients were excluded from the subgroup intervention trial, thus being unable to investigate an optional effect of exenatide on liver steatosis, as recent literature suggests
Vedtofte et al., (2020) [60]	Denmark	Randomized, placebo-controlled trial	82	Age: 38.8 years (range: 34.3–40.7)	Liraglutide (1.8 mg once daily)	52 weeks	Gestational diabetes mellitus	Ultrasound	Not reported	One-year’s liraglutide treatment had no effect on the presence of ultrasound-diagnosed NAFLD in overweight/obese nondiabetic women with pGDM, but reduced body weight and steatosis assessed by transient elastography with CAP	Not reported

Quality Assessment

**Figure 2:**
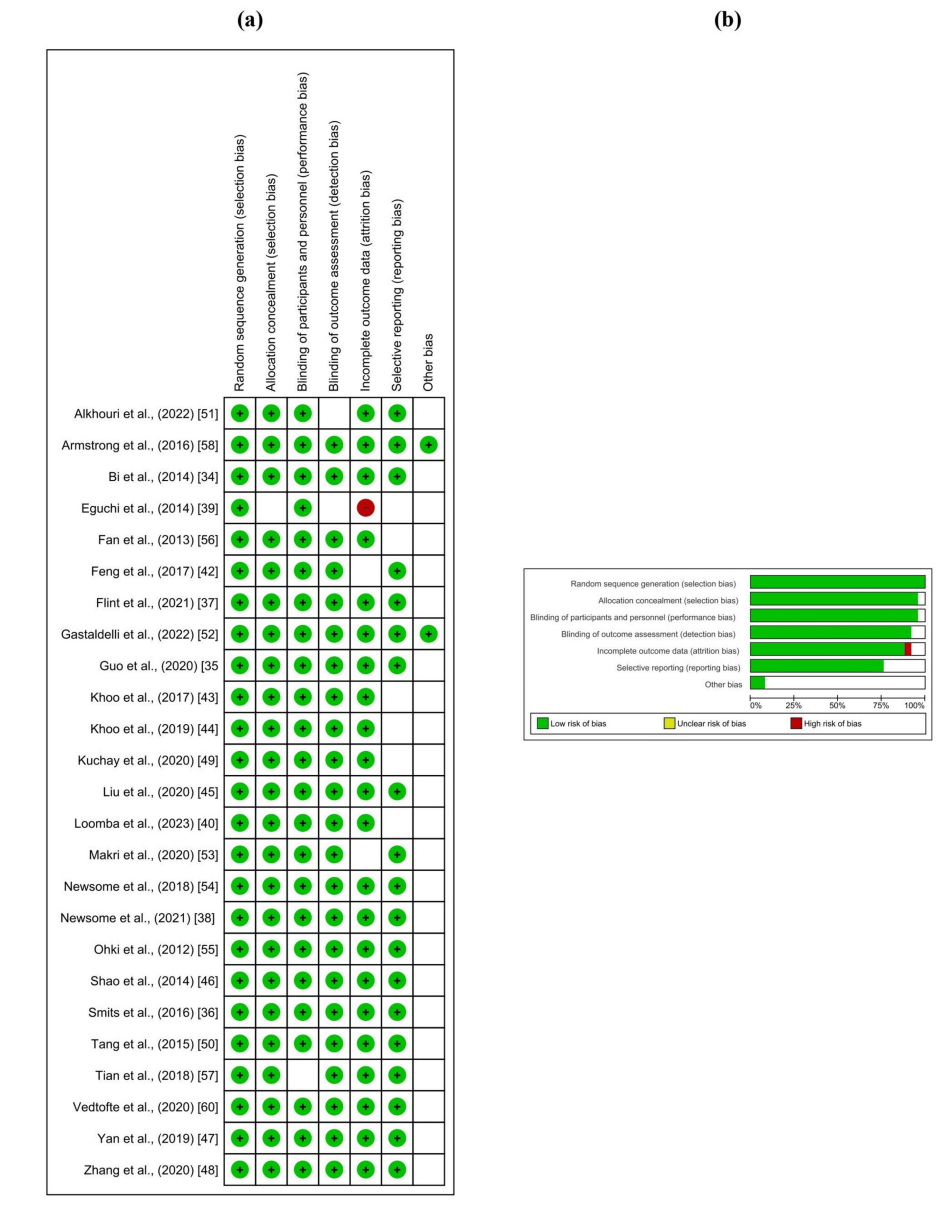
(a) Summary of the results of quality assessment of included studies based on the RoB2 tool. (b) Overall risk-of-bias assessment in systematic review studies. RoB2, Cochrane Risk of Bias tool

The quality appraisal of the 27 RCTs included in the systematic review, guided by the Risk of Bias (ROB2) assessment tool, reflects a commendably low risk of bias across key domains. The evaluation showed that most studies maintained low bias in random sequence generation, allocation concealment, and blinding of participants, which signifies rigorous study designs. Nevertheless, a minority of studies showed some concerns, particularly in the domain of blinding of outcome assessment, indicating areas where the risk of bias was unclear, potentially affecting the strength of the evidence. Overall, selective reporting was very well managed, with the vast majority of studies presenting low bias, underscoring the transparency and reliability of the reported findings. These results highlight the methodological soundness of the included studies, though the few instances of unclear risk remind us to interpret the findings with a degree of caution. Figure 2 illustrates the distribution of these biases in detail, providing a visual representation of the robustness of the study pool.

Outcomes

Under the umbrella of outcomes examined in the meta-analysis, various parameters were systematically examined to assess the efficacy and safety of GLP-1RAs in patients with MASLD. These outcomes were meticulously categorized into hepatic outcomes, cardiovascular outcomes, anthropometric measurements, and mortality, each representing key domains essential for evaluating the impact of GLP-1RAs.

Hepatic Outcomes

**Figure 3:**
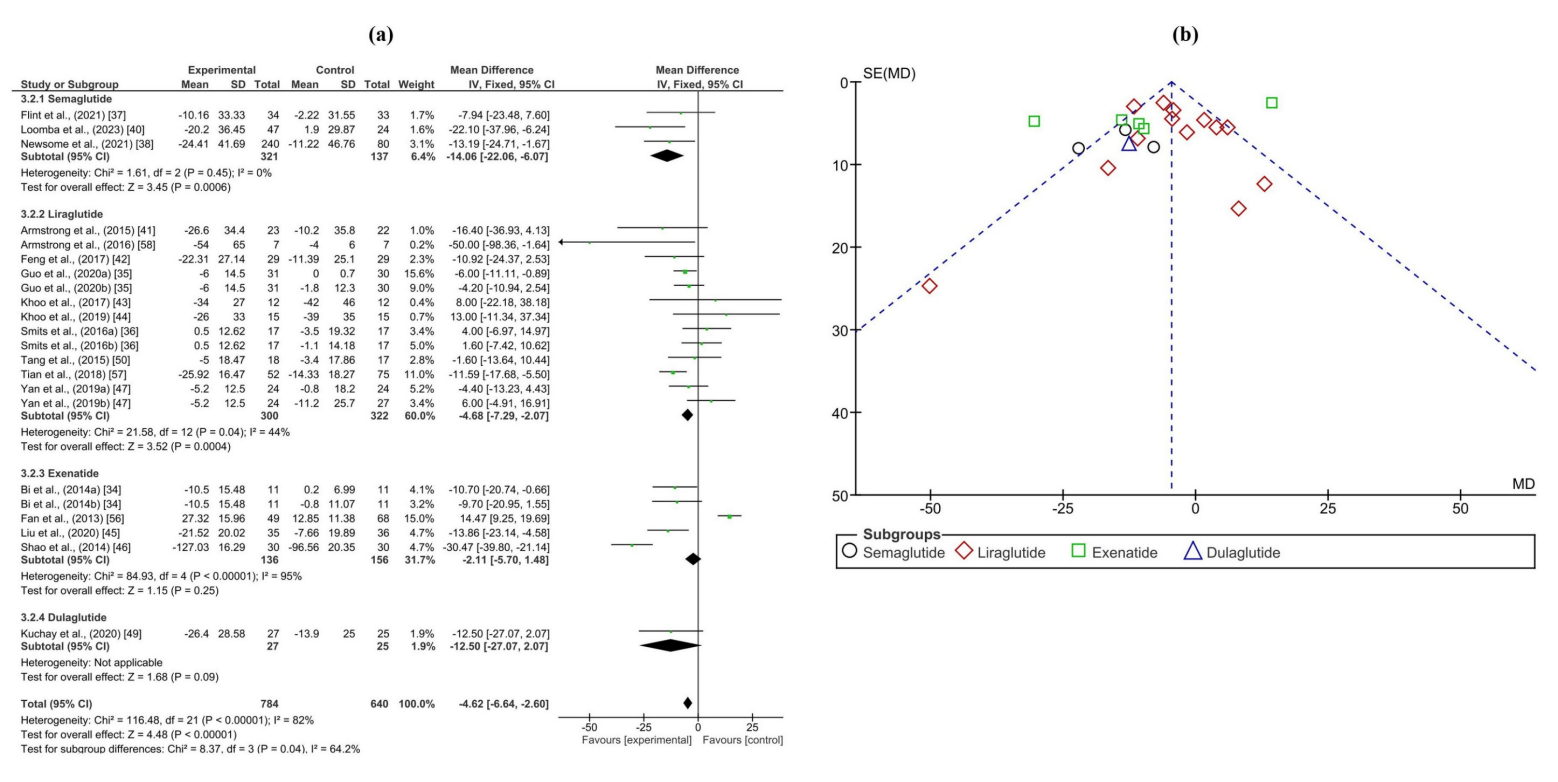
(a) Forest plot of intervention efficacy on ALT. (b) Funnel plot of research homogeneity for ALT. ALT, alanine aminotransferase

**Figure 4:**
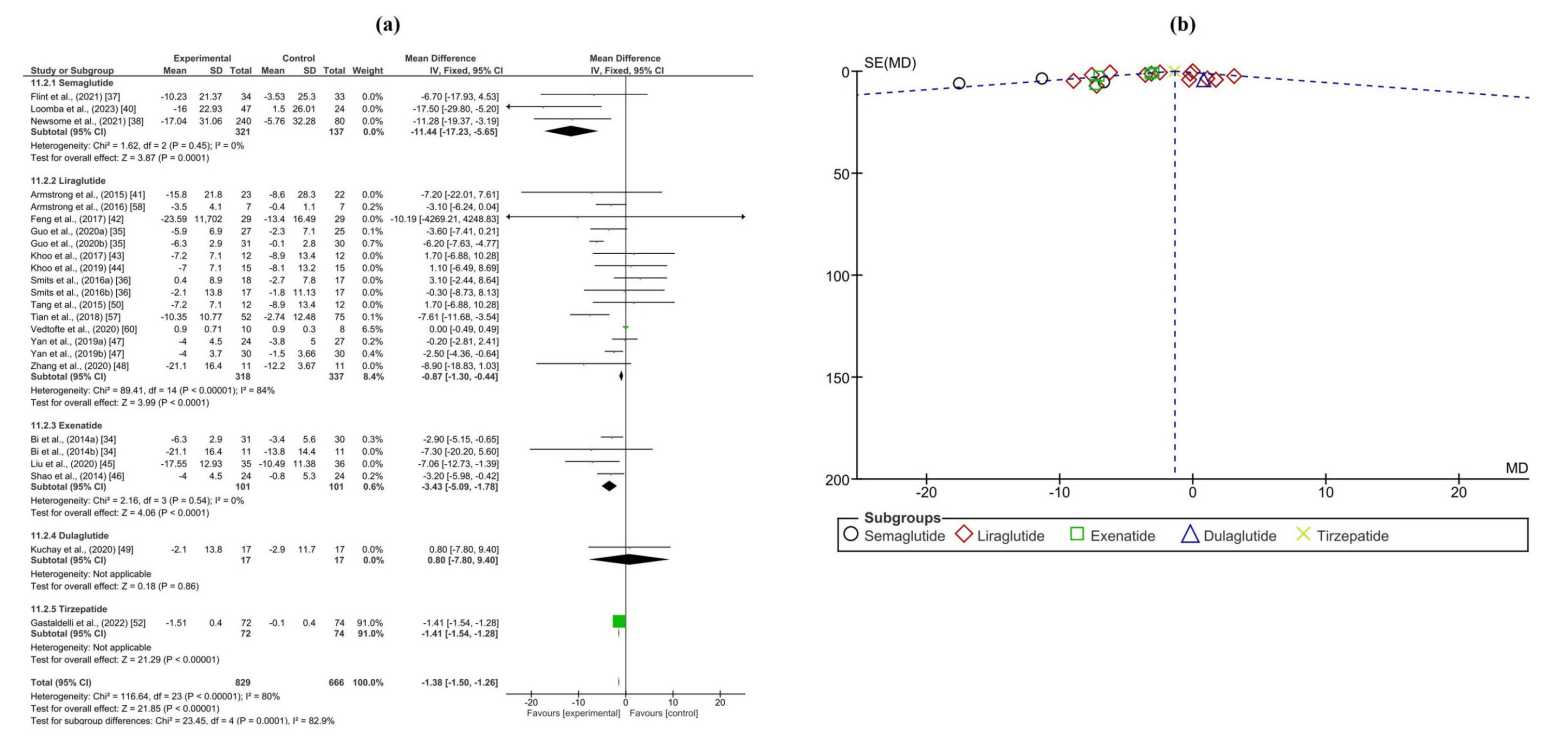
(a) Forest Plot of intervention efficacy on LFC. (b) Funnel plot of research homogeneity for LFC. LFC, liver fat content

The meta-analysis of hepatic outcomes encompassed several parameters including alanine aminotransferase (ALT), aspartate aminotransferase (AST), gamma-glutamyl transferase (GGT), liver fat content (LFC), and fibrosis-4 index (FIB-4) score. In the analysis of ALT levels, involving 784 patients and 640 controls across 18 trials, a significant reduction was observed in patients treated with GLP-1RAs, with a mean difference of -4.62 (-6.64, -2.60) (p < 0.0001). Similarly, the meta-analysis of AST levels, comprising 824 patients and 678 controls from 20 trials, revealed a statistically significant decrease with a mean difference of -0.21 (-0.38, -0.04) (p < 0.0001). Analysis of GGT levels, involving 235 patients and 220 controls across eight trials, demonstrated a significant decrease in the GLP-1RA group, with a mean difference of -0.71 (-0.94, -0.47) (p < 0.0001). Moreover, the meta-analysis of LFC, with 829 patients and 666 controls from 20 trials, exhibited a substantial reduction in patients receiving GLP-1RAs compared to controls, with a mean difference of -1.38 (-1.50, -1.26) (p < 0.0001). However, the FIB-4 score meta-analysis, including 117 patients and 121 controls from three trials, did not show a significant difference between the treatment and control groups, with a mean difference of -0.04 (-0.15, 0.08) (p=0.5). Forest and funnel plots depicting the results of these analyses are shown in Figures 3, 4 and Appendices C-E.

Cardiovascular Outcomes

**Figure 5:**
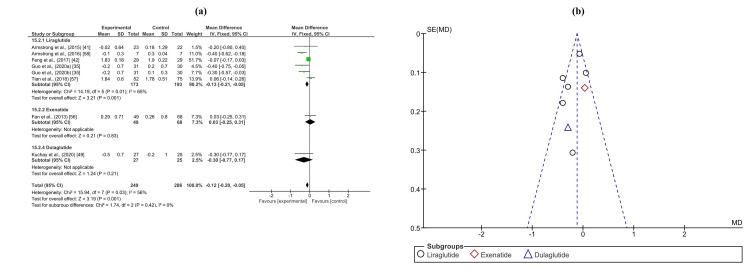
(a) Forest plot of intervention efficacy on triglyceride. (b) Funnel plot of research homogeneity for triglyceride.

The analysis of cardiovascular outcomes revealed several significant findings across various parameters. One trial investigated diastolic blood pressure, comprising 23 patients and 22 controls, showing no significant difference between groups, with a mean difference of -1.80 (-8.30, 4.70) (p = 0.59). However, in the meta-analysis of fasting blood sugar involving 274 patients and 291 controls from 10 trials, a significant decrease was observed in the GLP-1RA group compared to controls, with a mean difference of -0.2 (-0.38, -0.02) (p = 0.03). Similarly, the meta-analysis of HbA1c levels, with 466 patients and 360 controls from 12 trials, demonstrated a significant reduction in patients treated with GLP-1RAs, with a mean difference of -0.33 (-0.41, -0.25) (p < 0.0001). Analysis of high-density lipoprotein (HDL) levels, comprising 227 patients and 241 controls across seven trials, also showed a significant increase in the GLP-1RA group, with a mean difference of -0.07 (-0.09, -0.05) (p < 0.0001). Moreover, the meta-analysis of low-density lipoprotein (LDL) levels, involving 227 patients and 241 controls from seven trials, exhibited a significant decrease in patients receiving GLP-1RAs compared to controls, with a mean difference of -0.12 (-0.18, -0.05) (p = 0.0002). However, in the analysis of systolic blood pressure with 30 patients and 29 controls from two trials, and total cholesterol levels with 193 patients and 232 controls from three trials, no significant differences were found between the treatment and control groups (systolic blood pressure: mean difference -3.89 [-9.71, 1.92], p = 0.19; total cholesterol: mean difference -0.12 [-0.30, 0.05], p = 0.17). Conversely, the meta-analysis of triglyceride levels, comprising 249 patients and 286 controls across seven trials, showed a significant decrease in patients treated with GLP-1RAs compared to controls, with a mean difference of -0.12 (-0.20, -0.05) (p = 0.001). Figure 5 and Appendices F-L present forest and funnel plots illustrating these findings.

Mortality

The analysis of mortality revealed limited data, with only one trial conducted by Newsome et al. reporting mortality data [38]. In this trial, one death was reported in the semaglutide 0.2 mg group during the trial period, confirmed by the external adjudication committee as sudden cardiac death.

Anthropometric measurements

**Figure 6:**
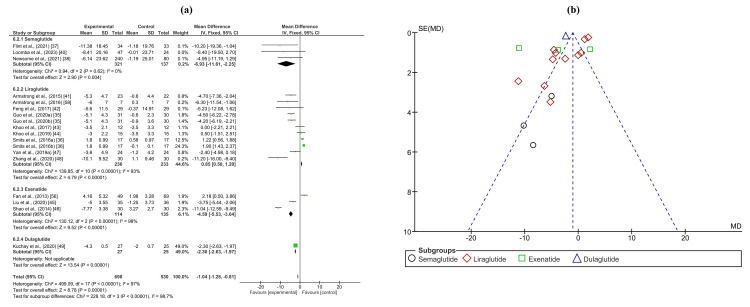
(a) Forest plot of intervention efficacy on body weight change. (b) Funnel plot of research homogeneity for body weight change.

The analysis of anthropometric measurements yielded significant results across various parameters. In the meta-analysis of body weight change, involving 698 patients and 530 controls from 16 trials, a significant decrease was observed in patients treated with GLP-1RAs compared to controls, with a mean difference of -1.04 (-1.28, -0.81) (p < 0.0001). Notably, liraglutide exhibited a positive mean difference in body weight change compared to the overall effect, with a mean difference of 0.85 (0.50, 1.20). Similarly, the meta-analysis of body mass index (BMI), comprising 321 patients and 357 controls across 11 trials, demonstrated a significant decrease in the GLP-1RA group compared to controls, with a mean difference of -0.74 (-0.86, -0.63) (p < 0.0001). Analysis of subcutaneous fat, with 217 patients and 221 controls from four trials, also revealed a significant reduction in patients receiving GLP-1RAs, with a mean difference of -5.43 (-5.61, -5.25) (p < 0.0001). Moreover, the meta-analysis of waist circumference, involving 267 patients and 265 controls from 10 trials, exhibited a significant decrease in the GLP-1RA group compared to controls, with a mean difference of -5.64 (-6.32, -4.97) (p < 0.00001). Forest and funnel plots depicting these findings are provided in Figure 6 and Appendices M, N.

Discussion

The primary aim of this systematic review and meta-analysis was to assess the efficacy and safety of GLP-1RAs in patients with MASLD. To the best of our knowledge, this is the most updated and comprehensive systematic review on the use of GLP-1RAs among MASLD patients. Our comprehensive analysis, encompassing 27 trials with a diverse patient population, yielded significant insights into the therapeutic potential of GLP-1RAs in this context. Across various parameters, including hepatic outcomes, cardiovascular markers, and anthropometric measurements, the findings of this review collectively highlight the favorable impact of GLP-1RAs. Notably, GLP-1RAs demonstrated consistent efficacy in improving hepatic function, as evidenced by significant reductions in key markers such as ALT, AST, GGT, and LFC. Moreover, improvements in cardiovascular risk factors, including fasting blood sugar, HbA1c levels, and lipid profiles, further support the potential benefits of GLP-1RAs in ameliorating metabolic dysregulation associated with MASLD. These findings collectively underscore the promising role of GLP-1RAs as a therapeutic option for patients with MASLD, addressing both hepatic and metabolic manifestations of the disease. 

The analysis of hepatic outcomes offers profound insights into the therapeutic potential of GLP-1RAs in patients with MASLD. The significant reductions observed in key liver enzymes, including ALT, AST, and GGT, not only signify improvements in liver function but also suggest a potential attenuation of hepatocellular injury and inflammation associated with MASLD [61]. These findings align with the known mechanisms of action of GLP-1RAs, which include reducing hepatic glucose production, enhancing insulin sensitivity, and exerting anti-inflammatory effects within the liver [62,63]. Moreover, the substantial decrease in LFC underscores the potential of GLP-1RAs to mitigate hepatic steatosis, a hallmark feature of MASLD and a precursor to more severe liver pathology. Various mechanisms in which this effect occurs have been reported in the literature [58,64,65], including evidence that shows the involvement of SIRT1 mediating the effect of exenatide on ameliorating hepatic steatosis [66]. SIRT1 plays a vital role in hepatic lipid metabolism by deacetylation of acetylated lysine residues on histones and various transcriptional regulators [67]. Despite this evidence, the lack of a significant difference in FIB-4 score between treatment and control groups in meta-analysis warrants careful consideration. While biochemical markers such as ALT and AST are indicative of hepatocellular injury, the FIB-4 score integrates both liver enzymes and patient demographics to estimate liver fibrosis [68], reflecting the dynamic interplay between inflammation, fibrogenesis, and metabolic perturbations in MASLD. Thus, the nonsignificant change in FIB-4 score suggests that while GLP-1RAs may effectively ameliorate hepatic injury and steatosis, their impact on the progression of fibrosis may be more nuanced and require longer-term follow-up and more research to elucidate fully. Nevertheless, the observed improvements in ALT, AST, GGT, and LFC hold profound clinical relevance, offering a potential avenue for therapeutic intervention in MASLD to preserve liver function and mitigate disease progression.

Furthermore, the analysis of cardiovascular outcomes offers compelling evidence of the multifaceted benefits of GLP-1RAs in patients with MASLD, extending beyond hepatic improvements to cardiovascular risk reduction. The significant improvements observed in fasting blood sugar and HbA1c levels following treatment with GLP-1RAs not only reflect enhanced glycemic control but also suggest a potential attenuation of systemic inflammation and insulin resistance, both of which are implicated in the pathogenesis of MASLD and its cardiovascular complications. Moreover, the favorable changes in lipid profiles, including increases in HDL cholesterol and decreases in LDL cholesterol and triglyceride levels, underscore the potential of GLP-1RAs to mitigate dyslipidemia, a major contributor to cardiovascular risk in MASLD patients. Previous research in the literature has shown that GLP-1RAs exert direct cardiovascular benefits through their actions on the cardiovascular system, including vasodilation, inhibition of platelet aggregation, and reduction of oxidative stress and inflammation [69,70]. These mechanisms contribute to improved endothelial function and vascular health, thus reducing the risk of cardiovascular events in patients with MASLD. These findings hold profound implications for the management of MASLD, as cardiovascular disease represents a significant comorbidity and contributor to morbidity and mortality in this population [71,72].

Moreover, our review analyzed mortality data to assess safety profiles of GLP-1RAs among MASLD patients. Overall, the analysis of mortality outcomes emphasizes the need for further research into the long-term safety profile of GLP-1RAs in patients with MASLD. While data on mortality were limited, the absence of significant safety concerns shown in qualitative data collected from trials aligns with the established favorable safety profile of GLP-1RAs in the literature [38].

Turning to anthropometric measurements, the significant reductions observed in body weight, BMI, subcutaneous fat, and waist circumference among patients treated with GLP-1RAs signify promising metabolic effects with potential implications for MASLD management. The observed reductions in body weight and adiposity suggest a multifaceted approach to improving metabolic health beyond hepatic outcomes. Reductions in adipose tissue are associated with improvements in insulin sensitivity, lipid profiles, and inflammatory markers, all of which are central to the pathogenesis of MASLD [73]. Furthermore, the significant decreases in waist circumference, a marker of visceral adiposity, are particularly noteworthy given its strong association with insulin resistance and cardiovascular risk [74,75].

Clinical Implications and Future Research

The findings of this systematic review and meta-analysis present compelling evidence for the potential clinical utility of GLP-1RAs in the management of MASLD. The observed improvements in hepatic outcomes, including significant reductions in liver enzymes and LFC, suggest a direct hepatoprotective effect of GLP-1RAs. These agents have been shown to modulate hepatic lipid metabolism, reduce hepatic inflammation, and promote liver regeneration, addressing key pathogenic mechanisms underlying MASLD. Additionally, the favorable changes in cardiovascular markers, such as improvements in glycemic control, lipid profiles, and blood pressure, highlight the broader cardiometabolic benefits of GLP-1RAs. By targeting systemic insulin resistance, dyslipidemia, and vascular dysfunction, GLP-1RAs offer a multifaceted approach to mitigating the cardiovascular risk burden in MASLD patients. Furthermore, the significant reductions in body weight and adiposity observed with GLP-1RA therapy not only contribute to metabolic improvements but also hold implications for reducing hepatic steatosis and inflammation. These multifactorial effects underscore the potential of GLP-1RAs to address the complex interplay between hepatic dysfunction, metabolic dysregulation, and cardiovascular risk in MASLD. As such, the findings underscore the importance of considering GLP-1RAs as a valuable addition to the therapeutic armamentarium for MASLD, warranting further investigation in clinical practice to optimize their role in disease management.

Moving forward, there is a critical need for further research to expand our understanding of the efficacy and safety profile of GLP-1RAs in patients with MASLD. Long-term studies and trials with larger sample sizes are essential to evaluate the sustained benefits and potential risks associated with GLP-1RAs in this population. Additionally, studies incorporating diverse patient populations and assessing real-world effectiveness are needed to validate the findings of this meta-analysis and inform clinical decision-making. Moreover, investigations into the optimal dosing regimens, treatment duration, and potential combination therapies involving GLP-1RAs are warranted to optimize their use in MASLD management. By addressing these knowledge gaps, future research endeavors hold the potential to further elucidate the role of GLP-1RAs in the comprehensive management of MASLD and to improve outcomes for affected patients.

Limitations

Several limitations of this systematic review and meta-analysis warrant consideration. Firstly, the limited number of trials reporting certain outcomes, such as mortality, restricts the robustness of our findings in these domains. Additionally, heterogeneity among included studies, stemming from variations in study design, patient populations, and intervention protocols, may introduce potential sources of bias and affect the generalizability of our results. Despite efforts to mitigate heterogeneity through subgroup analyses and sensitivity analyses, residual heterogeneity may still exist, influencing the interpretation of our findings. Furthermore, the possibility of publication bias, wherein studies with positive outcomes are more likely to be published, cannot be disregarded. Although we attempted to minimize publication bias by conducting a comprehensive literature search and including unpublished studies, the potential for bias remains inherent in meta-analyses of published literature. Recognizing these limitations is crucial for interpreting the findings of this study accurately and underscores the need for cautious interpretation and further research to validate our results and address these inherent limitations.

## Conclusions

The findings of this systematic review demonstrate promising clinical implications for the use of GLP-1RAs as a therapeutic option for addressing the complex pathophysiology of MASLD. By targeting hepatic dysfunction, metabolic abnormalities, and cardiovascular risk factors, GLP-1RAs offer a multifaceted approach to managing MASLD, with the potential to improve liver function, mitigate cardiovascular risk, and promote weight loss. However, it is essential to interpret those findings while considering the limitations of this study, including the limited number of trials reporting certain outcomes, heterogeneity among included studies, and potential publication bias. Further research, including long-term studies and trials with larger sample sizes, is warranted to validate our findings and optimize the use of GLP-1RAs in MASLD management. Nevertheless, the findings of this study highlight the importance of considering GLP-1RAs as a valuable addition to the therapeutic armamentarium for MASLD, offering new avenues for addressing this increasingly prevalent and burdensome liver disease.

## Figures and Tables

**Figure 7:**
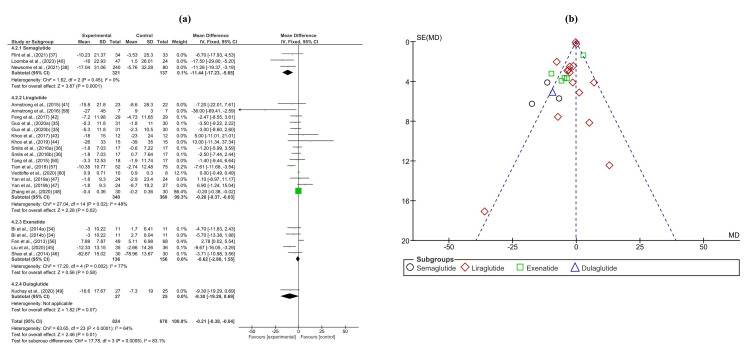
(a) Forest plot of intervention efficacy on AST. (b) Funnel plot of research homogeneity for AST. AST, aspartate aminotransferase

**Figure 8:**
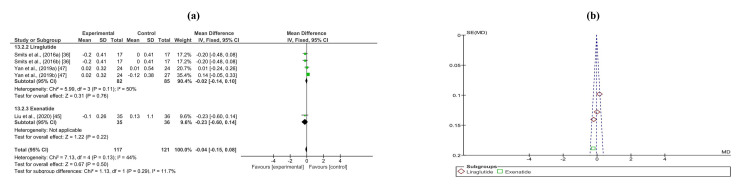
(a) Forest plot of intervention efficacy on FIB-4 score. (b) Funnel plot of research homogeneity for FIB-4 score. FIB-4, fibrosis-4 index

**Figure 9:**
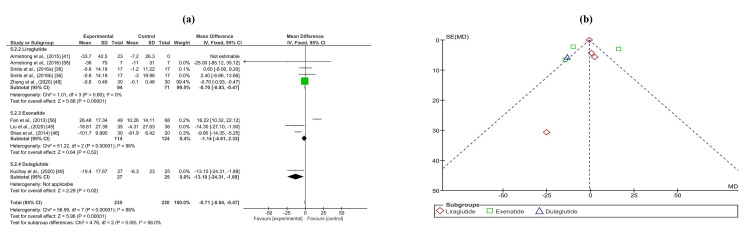
(a) Forest plot of intervention efficacy on GGT score. (b) Funnel plot of research homogeneity for GGT score. GGT, gamma-glutamyl transferase

**Figure 10:**
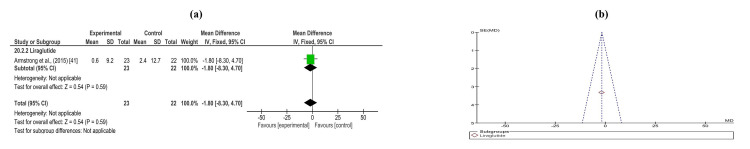
(a) Forest plot of intervention efficacy on diastolic blood pressure. (b) Funnel plot of research homogeneity for diastolic blood pressure.

**Figure 11:**
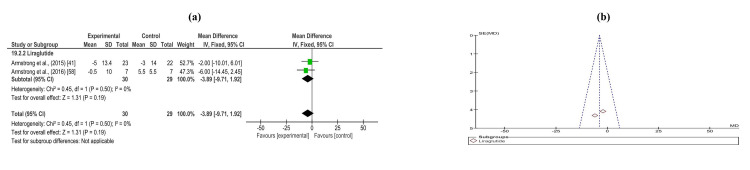
(a) Forest plot of intervention efficacy on systolic blood pressure. (b) Funnel plot of research homogeneity for systolic blood pressure.

**Figure 12:**
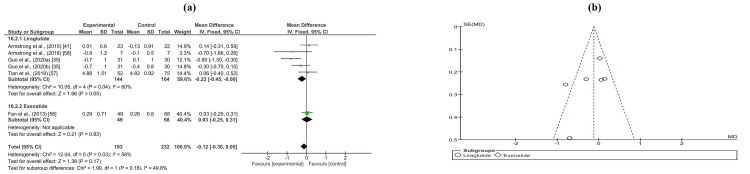
(a) Forest plot of intervention efficacy on total cholesterol. (b) Funnel plot of research homogeneity for total cholesterol.

**Figure 13:**
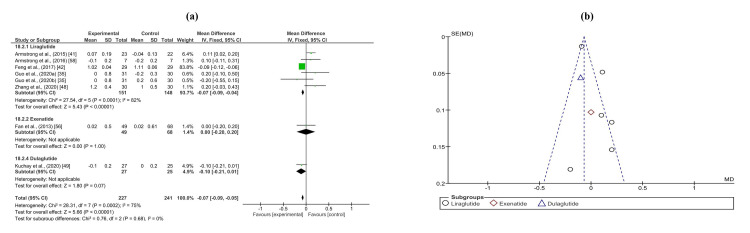
(a) Forest plot of intervention efficacy on HDL levels. (b) Funnel plot of research homogeneity for HDL levels. HDL, high-density lipoprotein

**Figure 14:**
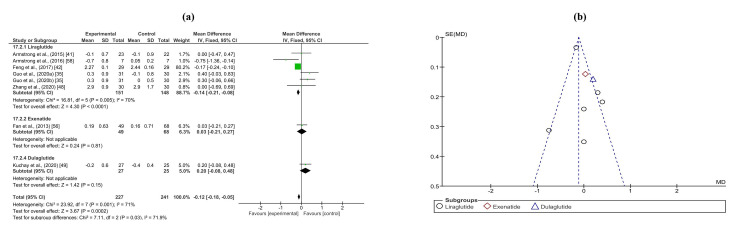
(a) Forest plot of intervention efficacy on LDL levels. (b) Funnel plot of research homogeneity for LDL levels. LDL, low-density lipoprotein

**Figure 15:**
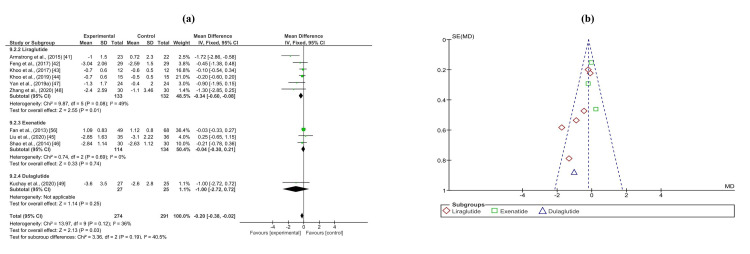
(a) Forest plot of intervention efficacy on fasting blood sugar. (b) Funnel plot of research homogeneity for fasting blood sugar levels.

**Figure 16:**
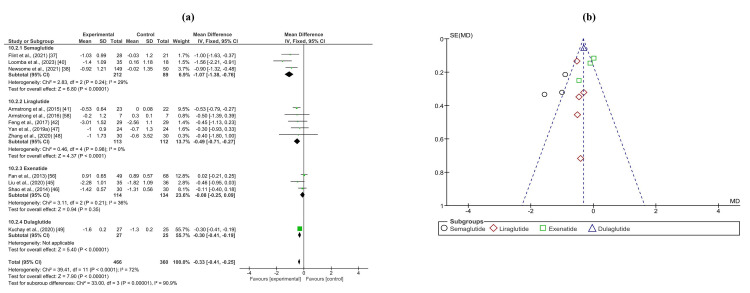
(a) Forest plot of intervention efficacy on HbA1c. (b) funnel plot of research homogeneity for HbA1c.

**Figure 17:**
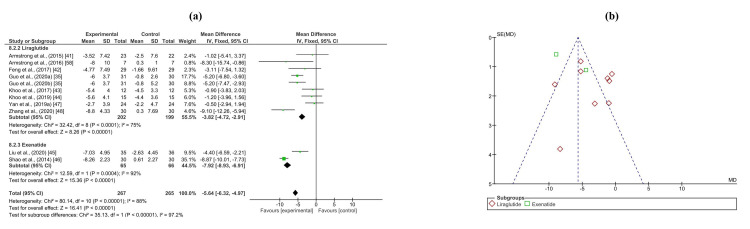
(a) Forest Plot of intervention efficacy on waist circumference. (b) Funnel plot of research homogeneity for waist circumference.

**Figure 18:**
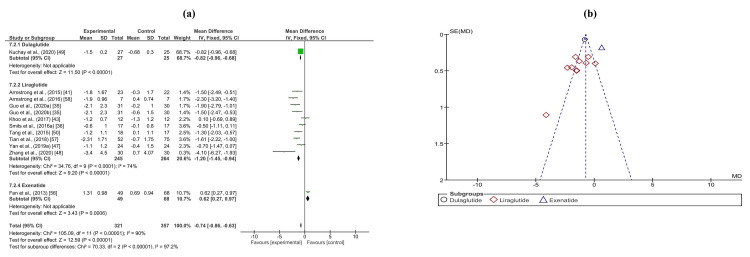
(a) Forest plot of intervention efficacy on BMI. (b) funnel plot of research homogeneity for BMI. BMI, body mass index

## Appendices

 Appendix A

**Table 2:** Literature search keywords for different databases used in the search

Search keywords
1) Population of interest: GLP-1 Receptor Agonists Users with/without MASLD ("GLP-1 Receptor Agonists" [MeSH] OR "GLP-1" [Title/Abstract] OR "Glucagon-Like Peptide-1" [Title/Abstract] OR "Exenatide" [Title/Abstract] OR "Liraglutide" [Title/Abstract] OR "Semaglutide" [Title/Abstract] OR "Dulaglutide" [Title/Abstract]) AND ("Metabolic Dysfunction-Associated Steatotic Liver Disease" [MeSH] OR "MASLD" [Title/Abstract] OR "Non-Alcoholic Steatotic Liver Disease" [Title/Abstract] OR "fatty liver" [Title/Abstract] OR "hepatic steatosis" [Title/Abstract] OR "NAFLD" [Title/Abstract] OR “Non-alcoholic Fatty Liver Disease" [MeSH])
2) Outcomes: Benefits and Risks in Clinical Trials ("Clinical Trials" [MeSH] OR "Randomized Controlled Trial" [Title/Abstract] OR "RCT" [Title/Abstract] OR "Meta-analysis" [Title/Abstract] OR "Systematic review" [Title/Abstract]) AND ("Efficacy" [Title/Abstract] OR "Effectiveness" [Title/Abstract] OR "Safety" [Title/Abstract] OR "Adverse Effects" [Title/Abstract] OR "Side Effects" [Title/Abstract] OR "Risk Assessment" [Title/Abstract] OR "Treatment Outcome" [Title/Abstract])

Appendix B 

**Table 3:** Eligibility criteria for studies upon screening the search results obtained. GLP-1, glucagon-like peptide-1; MASLD, metabolic dysfunction-associated steatotic liver disease; RCT, randomized controlled trial

Inclusion criteria	Exclusion criteria
Study conducted in English	Paper not peer-reviewed
Primary outcome reports efficacy, safety, or treatment outcomes of GLP-1 receptor agonists in patients with or without MASLD	Full text paper not available and/or written in non-English language
Study design: RCTs	Study design being a review of the literature (e.g., scoping, systematic, narrative, or other reviews), case reports, case series, or studies conducted in animal models
Primary outcome reporting among MASLD adult patients (older than 18 years)	Pediatric populations
Full-text paper available	Studies lacking relevant data on GLP-1 receptor agonists in MASLD
Peer-reviewed prior to final publication	Studies focusing on diseases or conditions unrelated to MASLD or not involving GLP-1 receptor agonists

Appendix C 

Appendix D 

Appendix E 

Appendix F 

Appendix G 

Appendix H 

Appendix I 

Appendix J 

Appendix K 

Appendix L 

Appendix M 

Appendix N 
